# Insecticide-impregnated dog collars for the control of visceral leishmaniasis: evaluation of the susceptibility of field *Lutzomyia longipalpis* populations to deltamethrin

**DOI:** 10.1186/s13071-024-06474-4

**Published:** 2024-11-15

**Authors:** Mayra de Sousa Félix de Lima, Rafaella Albuquerque e Silva, Douglas de Almeida Rocha, Gabriela de Oliveira Mosqueira, Rodrigo Gurgel-Gonçalves, Marcos Takashi Obara

**Affiliations:** 1 Post Graduate Program in Tropical Medicine, School of Medicine, University of Brasília, Brasilia, Distrito Federal Brazil; 2 Ministry of Health, General Coordination of Zoonoses and Vector-Borne Diseases Surveillance, Federal District, Brasilia, Brazil; 3 Independent Researcher, Brasília, Brazil

**Keywords:** Sand flies, Control, Dogs, Resistance, Bioassays

## Abstract

**Background:**

Visceral leishmaniasis (VL) is a zoonotic disease caused by *Leishmania infantum* and transmitted by the sand fly *Lutzomyia longipalpis*. Dogs are the major domestic reservoir of *L. infantum*. To prevent the spread of the disease, dog collars impregnated with 4% deltamethrin have been effectively used in VL endemic areas. However, this approach may contribute to the emergence of insecticide resistance in sand flies. Therefore, it is important to characterize the susceptibility of different populations of *Lu. longipalpis* to deltamethrin in areas where insecticide-impregnated dog collars are used.

**Methods:**

Six field sand fly populations from Brazil were exposed to deltamethrin in CDC bottle bioassays at the diagnostic doses (DD) of 21.9 μg/bottle and 30 μg/bottle. For the dose–response (DR) experiments, doses of 1, 3, 5, 7, 9 and 11 μg/bottle of deltamethrin were used to impregnate bottles; control group bottles were impregnated with acetone only. Each bottle contained an average of 20 sand flies, both male and female, and they were exposed to either deltamethrin or acetone for 60 min.

**Results:**

Based on the DD of 21.9 μg/bottle, three populations were susceptible to deltamethrin. In contrast, two populations collected from the states of Ceará and Minas Gerais exhibited mortality rates of 94.9% and 95.7%, indicating possible resistance, and one population from the state of Ceará showed resistance, with a mortality rate of 87.1%. At the DD of 30 μg/bottle, two populations from the states of Ceará and Piauí showed possible resistance, while the other four populations were susceptible. The resistance ratio (RR_50_) ranged from 2.27 to 0.54, and RR_95_ ranged from 4.18 to 0.33, indicating a low resistance intensity.

**Conclusions:**

This study established a DD for *Lu. longipalpis* using the CDC bottle bioassay. We found that *Lu. longipalpis* populations in three Brazilian states where insecticide-impregnated dog collars were used for VL control were susceptible to deltamethrin. However, one population in Ceará State was classified as resistant to deltamethrin. These results contribute to the current knowledge on sand fly resistance and surveillance, and highlight the need for a better understanding of the resistance mechanisms of *Lu. longipalpis* in areas where insecticide-impregnated dog collars have been widely used.

**Graphical Abstract:**

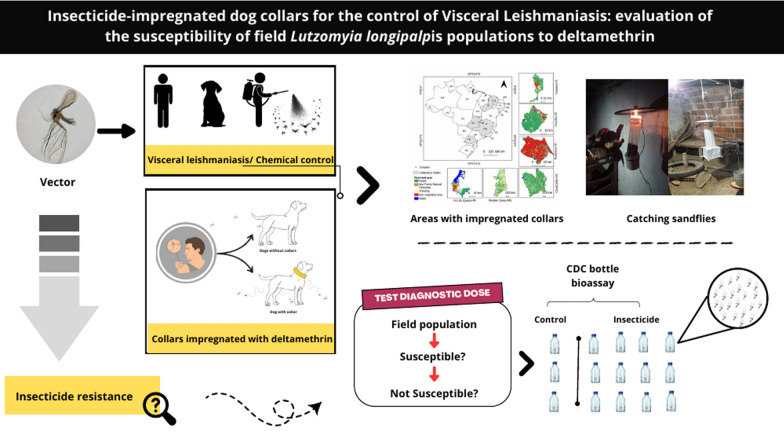

## Background

Visceral Leishmaniasis (VL) is a tropical zoonosis of major public health importance that is considered to be a neglected tropical disease [1]. This disease primarily affects children under the age of 5 years and adults older than 50 years, as well as those with comorbidities and immunocompromised conditions [2, 3]. The main mode of transmission to humans is through the bite of female sand flies infected with *Leishmania* species*. Lutzomyia longipalpis* (Lutz & Neiva, 1912) is the main vector of *Leishmania infantum* in the Americas [2, 4]. The WHO estimates that between 50,000 and 90,000 new cases of VL are reported annually. In the Americas, 69,665 new cases of VL were registered between 2001 and 2022, with an average of 4322 confirmed cases annually [5]. In Brazil, VL has spread throughout the country due to urbanization processes that have occurred since the 1980s [6]. Between 2001 and 2022, approximately 67,384 cases of VL were confirmed, with the majority occurring in the Northeast region of Brazil [7].

The prevention and control of VL have been primarily based on vector control due to the lack of safe and efficient vaccines [8–11]. In Brazil, the first attempts at chemical control of sand flies were made in the 1950s with the spraying of dichlorodiphenyltrichloroethane (DDT) in houses [12–14]. Falcão and colleagues [15] pioneered the use of pyrethroids, evaluating the efficacy of deltamethrin to control the *Lu. longipalpis* species. Since then, pyrethroids have been the primary compounds used to control sand flies, mosquitoes and kissing bugs; however, this method can lead to the selection of pyrethroid-resistant insect populations.

Insecticide resistance in sand flies can pose a threat to the effectiveness of VL control programs. Currently, there are records of *Phlebotomus argentipes* populations that are resistant to DDT, permethrin and deltamethrin [16–20]. Studies have shown resistance of *Phlebotomus papatasi* to various insecticides, such as DDT, permethrin and lambda cyhalothrin [16–22]. Additional studies have demonstrated the resistance of *Phlebotomus sergenti* to DDT [16, 23]. Until now, *Lu. longipalpis* populations have not shown resistance to insecticides [24–27].

Collars impregnated with the 4% insecticide deltamethrin protect the dog from the bite of female sand flies, thereby reducing the transmission of VL in dogs [28–31]. In light of the expansion of VL cases in the Americas [5], there is a high probability of sand fly resistance to the insecticides used to control VL [24]. However, limited knowledge is available on the potential for sand fly resistance to collars impregnated with deltamethrin. It is important to note that information on the occurrence of resistance in areas where insecticide-impregnated dog collars are used is scarce. Consequently, monitoring the susceptibility of *Lu. longipalpis* to insecticides, particularly in areas where insecticide-impregnated dog collars are used, is crucial for the improvement of vector control measures. The aim of this article was to characterize the susceptibility profile of different populations of *Lu. longipalpis* to deltamethrin in areas where insecticide-impregnated dog collars are used for VL control. We performed CDC vial bioassays to assess the susceptibility of field populations of *Lu. longipalpis* in Brazil to deltamethrin in areas treated with insecticide-impregnated dog collars. The results demonstrated that the majority of *Lu. longipalpis* populations tested in the study exhibited susceptibility to deltamethrin, except for those collected in Ceará State.

## Methods

### Reference population

The reference population (laboratory reference strain [LRS]) consisted of *Lu. longipalpis* sand flies from Jacobina in the Brazilian state of Bahia. The insects were reared in the Laboratório de Bioquímica e Fisiologia de Insetos of the Instituto Oswaldo Cruz in Rio de Janeiro. This insecticide-susceptible reference population has been bred and maintained in a colony for at least 5 years, with no input of external material. The insects are kept in semi-controlled conditions of temperature and humidity (28 ± 2 °C and 65 ± 10% relative humidity) under a 12/12-h light/dark photoperiod.

### Sand fly sampling

**Fig. 1:**
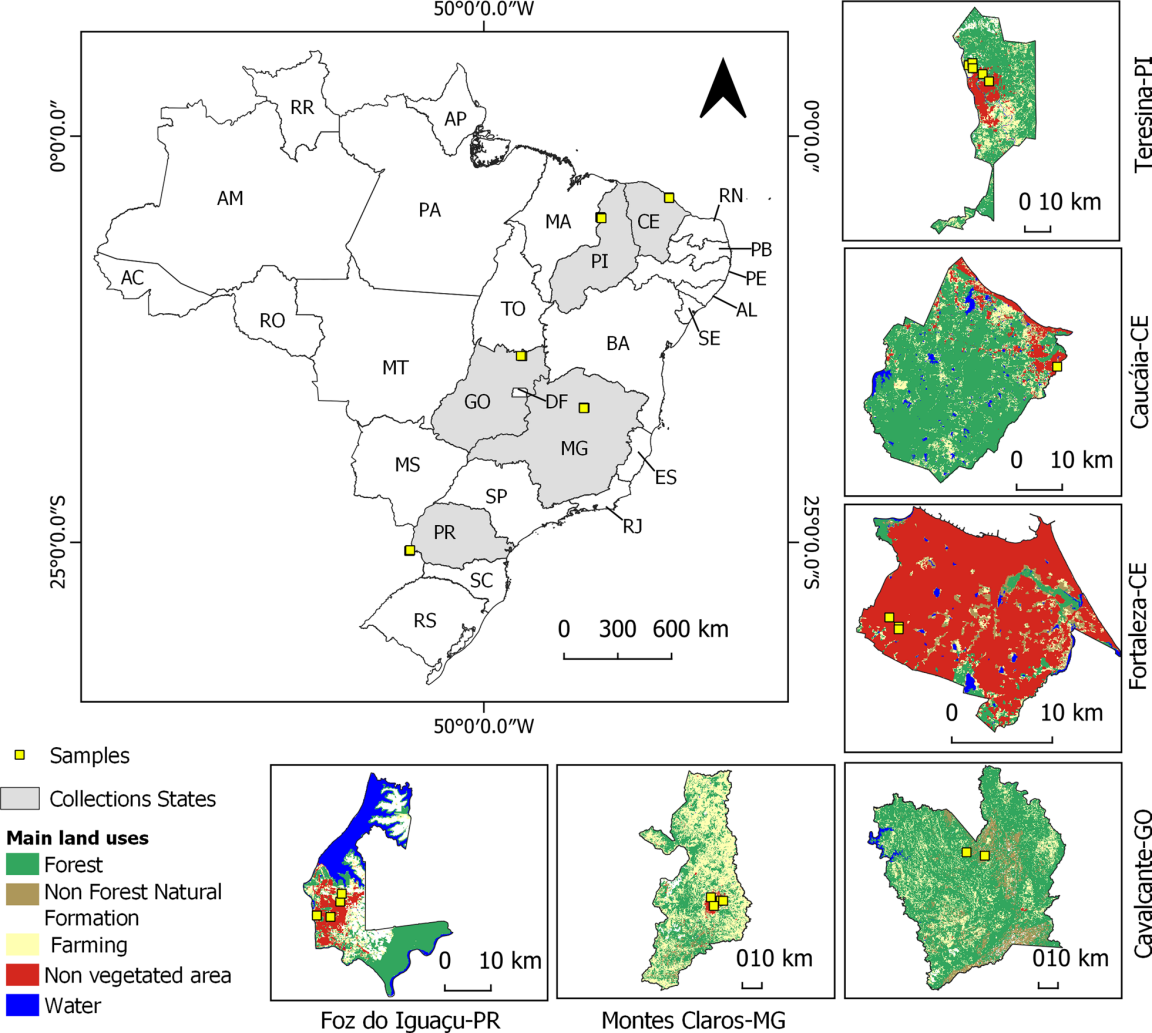
Map of Brazil showing the states (gray shading) and municipalities (featured along the edge) where the collections and bioassays with CDC bottles were carried out to assess the susceptibility of *Lutzomyia longipalpis* exposed to the insecticide deltamethrin in 2023. CE, Ceará State; GO, Goiás State; PI, Piauí State; MG, Minas Gerais State; PR, Paraná State. Source: MapBiomas (2023) (https://brasil.mapbiomas.org/en/)

For this study, we captured sand flies in six municipalities: Foz do Iguaçu, Paraná State (25°32′49″ S, 54°35′18″ W), Fortaleza, Ceará State (3°43′6″ S, 38°32′36″ W), Caucaia, Ceará State (3°44′4″ S, 38°39′23″ W), Teresina, Piauí State (5°5′21″ S, 42°48′6″ W), Montes Claros, Minas Gerais State (16°44′13″ S, 43°51′53″ W) and Cavalcante, Goiás State (13°47′51″ S, 47°27′20″ W) (Fig. 1). We selected these municipalities based on the following specifications: (i) high transmission of VL; (ii) long-term use by residents of insecticide-impregnated dog collars; (iii) presence of a specific VL control program; and (iv) households with *Leishmania*-infected dogs. The study areas were selected based on the VL risk stratification criteria established for VL risk stratification by the Brazilian Ministry of Health and the operational guidelines for the implementation of insecticide-impregnated collars (4% deltamethrin) in designated priority municipalities [32]. In addition, data on collection sites and canine positivity rates were gathered from the municipal health departments.

We captured sand flies using HP and/or CDC light traps, which were installed in the peridomicile of the houses, such as chicken coops, for 3 consecutive nights (from 5:00 p.m. to 8:00 a.m.). The following day, the traps were collected in the morning, and the sand flies were transported in polystyrene boxes containing moistened paper towels. In the municipal entomology laboratories, the traps were separated into two groups, those which contained sand flies and those which did not. Finally, the sand flies collected were transferred to larger entomological cages (30 × 30 cm), providing more storage space and consequently increasing the comfort of the insects, and maintained in the dark, with a black cloth covering the cage. This procedure was implemented for 1 h to facilitate the insects’ acclimation until the biological bioassays were conducted.

### Bioassays

The bioassays employed pyrethroid deltamethrin (C_22_H_19_Br_2_NO_3_; purity 99.6%; Bayer AG, Leverkusen, Germany). To prepare the stock solution, 10.1 mg of technical-grade insecticide (powder) was weighed and diluted in 1 ml of acetone (PA-Dynamics, Cheswick, PA, USA). A second solution was prepared from the stock solution to obtain a 0.5 mg/ml concentration for the experiments. Subdoses of 1, 3, 5, 7, 9, 11 μg/bottle deltamethrin/bottle were then prepared for the dose–response (DR) experiments to determine the diagnostic dose (DD), which is defined as the dose of insecticide per bottle that kills 99% of susceptible insects in a given period. A DR bioassay was conducted using a susceptible population to estimate the DD. Six different concentrations of insecticides were administered (1, 3, 5, 7, 9 and 11 μg/bottle), and the dose that caused 100% of the insects to die in the shortest reading time was considered the DD. The DD was established by multiplying the dosage required to kill 99% of insects LD_99_by 1. The DD determined here was 21.9 μg/bottle. The DD of 30 μg/bottle was recommended by Delinger et al. [33] for *Lu. longipalpis* populations exposed to the pyrethroid deltamethrin. This dose was determined from a reference colony at the Walter Reed Army Institute of Research (WRAIR; Silver Spring, MD, USA), and the bioassays were conducted at Utah State University (Logan, UT, USA).

Wheaton glass vials (250 ml) were impregnated with 1 ml of deltamethrin, while the control vials were impregnated with 1 ml of acetone only. The vials were labeled with the name of the used insecticide, the concentration and the date of the test. This vial impregnation process was carried out evenly on all sides of the vials, including the top and bottom, by rotating the container and carefully observing the vial. After vial impregnation, the lids were removed to prevent condensation. The vials were left uncovered for 1 h to allow the acetone to evaporate. Once dry, the vials were capped and stored in 29-l plastic boxes covered with a black cloth to protect them from light.

The CDC bottle bioassay described by the WHO [34] was used for all experiments.

 For the DD experiments, doses of 21.9 and 30.0 μg/bottle were administered to approximately 20 sand flies (males and females) per bottle from the field collection. The sand flies were removed from the cages using a Castro catcher. Fifteen bottles were used, with three bottles designated for the control group.

 The DR bioassays were performed in the field. Twenty-one bottles were used at this stage, with three bottles for each concentration (1, 3, 5, 7, 9 and 11 μg/bottle), totaling 18 bottles with insecticide and three controls (treated with acetone only). After introducing the sand flies into the control bottles and the bottles with each concentration of insecticide, we recorded the time for each bottle, and subsequently assessed mortality at 10-min intervals, with both dead and alive sand flies being recorded. Approximately 20 sand flies (males and females) were exposed to each dose. We used a Castro catcher and a small funnel to introduce the sand flies into each bottle, recording from minute zero until 60 min of exposure had elapsed. The experiment began by introducing sand flies into the control bottles, which were promptly closed and positioned horizontally. To prevent contamination, a Castro catcher and separate funnel were used to insert the sand flies into the control group. After the experiment, the sand flies were transferred to 250-ml plastic jars with protective cotton mesh containing a 10% sugar solution. Mortality rates were taken within 24 h by a single researcher. Following the mortality assessment, the sand flies were stored in Eppendorf tubes containing isopropyl alcohol to be preserved until they were identified at the species level. Each sand fly in an Eppendorf tube was identified by bioassay assessment and municipality. Mortality assessment was based on the criteria described by Rocha et al. [35]: (i) inability to fly in a coordinated manner; (ii) inability to stand; and (iii) brief standing and flying ability followed by immediate falling. The resistance assessment criteria were obtained from WHO [34]. The criteria are as follows: (i) mortality of ≥ 98% indicates susceptibility; (ii) mortality ≥ 90% but < 98% suggests the possibility of resistance; and (iii) mortality of < 90% suggests established/confirmed resistance. The RR_50_ and RR_95_ (resistance ratio) were calculated using the 50% lethal dose (LD_50_) and LD_95_ estimated for each population, respectively. The resistance ratio was determined in accordance with the WHO [36] guidelines. When the resistance ratio is ≤ 5, the field population is considered to be susceptible; when the resistance ratio is between 5 and 10, the mosquitoes are considered to be moderately resistant; and when the resistance ratio is > 10, the mosquitoes are considered to be highly resistant.

 The sand flies were then transported to the Laboratório de Parasitologia Médica e Biologia de Vetores at the University of Brasília for taxonomic identification. The male and female specimens were mounted and identified following the methods of Forattini [37] using the Galati taxonomic key [38] and the Lutzodex app [39]. The genera were abbreviated according to Marcondes [40].

### Statistical analysis

The mortality data of the *Lu. longipalpis* populations was used to estimate the concentration of insecticide that kills 50% (LD_50_) 95% (LD_95_) and 99% (LD_99_) of the samples studied. The LD_50_, LD_95_ and LD_99_ for each population were calculated using Probit analysis [41] and the Basic Probit Analysis and Polo Plus programs [42]. Lethal doses were expressed in micrograms per bottle (μg/bottle). The slope of the DR curve, which represents the homogeneity of the population and indicates the progression of resistance and genotypic variation in tolerance to an insecticide, was estimated using the GraphPad Prism program version 5.0 (GraphPad Software, San Diego, CA, USA). Lower values of the slope indicate more heterogeneous populations and, consequently, a greater probability of resistance selection. Resistance ratios were calculated by dividing the LD_50_ of the field population by the LD_50_ of the susceptible strain. The resistance ratio indicates the magnitude of the population's resistance to the insecticide.

## Results

### *Lutzomyia longipalpis* was the most captured species in the study areas

Among the 4094 sand flies captured in this study, *Lu. longipalpis* species was the most common sand fly species, accounting for 94% of specimens. The remaining species were *Migonemyia migonei* (0.8%), *Evandromyia lenti* (0.6%), *Evandromyia sallesi* (0.3%), and *Nyssomyia whitmani* (0.1%).

### Bioassays with six populations of *Lu. longipalpis* from areas using insecticide-impregnated dog collars

The LD_99_ for the reference population of *Lu. longipalpis* was 21.9 μg/bottle, and the lethal time for 100% mortality was 60 min of exposure for the dose of 11 μg/bottle. Bioassays of CDC bottles impregnated with the DD of 21.9 μg/bottle were performed on 1196 sand flies collected in the six areas where impregnated dog collars were used for VL control. The results showed that most *Lu. longipalpis* populations from the municipalities of Foz do Iguaçu, Cavalcante and Teresina were susceptible to deltamethrin. However, the populations from Ceará State (Caucaia and Fortaleza) and Minas Gerais State (Montes Claros) showed mortality rates ranging from 87.1% to 95.7%, suggesting resistance and possible resistance (Table 1).

**Table 1:** Mortality rate of sand fly populations exposed to the diagnostic dose of 21 μg deltamethrin/bottle in CDC bottle bioassays in 2023

Population	*N*	Mortality (%) following exposure to deltamethrin (21.9 μg/ bottle)							Classification
		Time after initial exposure							
		10 min	20 min	30 min	40 min	50 min	60 min	24 h	
CAU	201	8.9	15.9	24.8	36.8	48.7	69.6	87.1	Resistance
FOR	219	6.8	13.2	21.9	30.1	55.3	64.8	94.9	Possible resistance
MOC	163	14.1	25.1	54.6	87.7	92.1	100	95.7	Possible resistance
FOZ	166	4.2	11.4	27.7	51.2	68.1	85.5	98.2	Susceptibility
CAV	247	14.1	30.7	85.8	93.9	96.3	99.1	99.2	Susceptibility
TER	200	11.1	19.5	44.1	72.1	80.1	92.5	99.5	Susceptibility

*CAU* Caucaia, Ceará State,* CAV* Cavalcante, Goiás State,* FOR* Fortaleza, Ceará State,* FOZ* Foz do Iguaçu, Paraná State,* MOC* Montes Claros, Minas Gerais State,* TER* Teresina, Piauí State

For the DD of 30 μg/bottle, approximately 1124 sand flies were used in the CDC bottle bioassays. The sand fly populations from Caucaia and Teresina showed mortality rates of 95.5% and 95.8%, respectively. These data suggest the possibility of resistance to deltamethrin in these populations (Table 2).

**Table 2:** Mortality rate of sand fly populations exposed to the diagnostic dose of 30 μg/bottle of deltamethrin in CDC bottle bioassays in 2023

Population	*N*	Mortality (%) exposure with deltamethrin (30 μg/ bottle)							Classification
		Time after initial exposure							
		10 min	20 min	30 min	40 min	50 min	60 min	24 h	
CAU	173	4.1	16.2	41.6	64.7	82.1	95.5	95.3	Possible resistance
TER	191	9.9	35.1	68.5	89.5	95.8	97.9	95.8	Possible resistance
FOZ	160	6.2	17.5	46.5	77.5	95.1	95.1	98.7	Susceptibility
MOC	153	19.6	44.4	98.1	99.3	99.9	100	99.3	Susceptibility
CAV	226	24.7	75.6	97.7	100	100	100	99.6	Susceptibility
FOR	221	9.9	19.9	37.5	50.2	68.7	78.7	99.9	Susceptibility

*CAU* Caucaia, Ceará State,* CAV* Cavalcante, Goiás State,* FOR* Fortaleza, Ceará State,* FOZ* Foz do Iguaçu, Paraná State,* MOC* Montes Claros, Minas Gerais State,* TER* Teresina, Piauí State

**Fig. 2:**
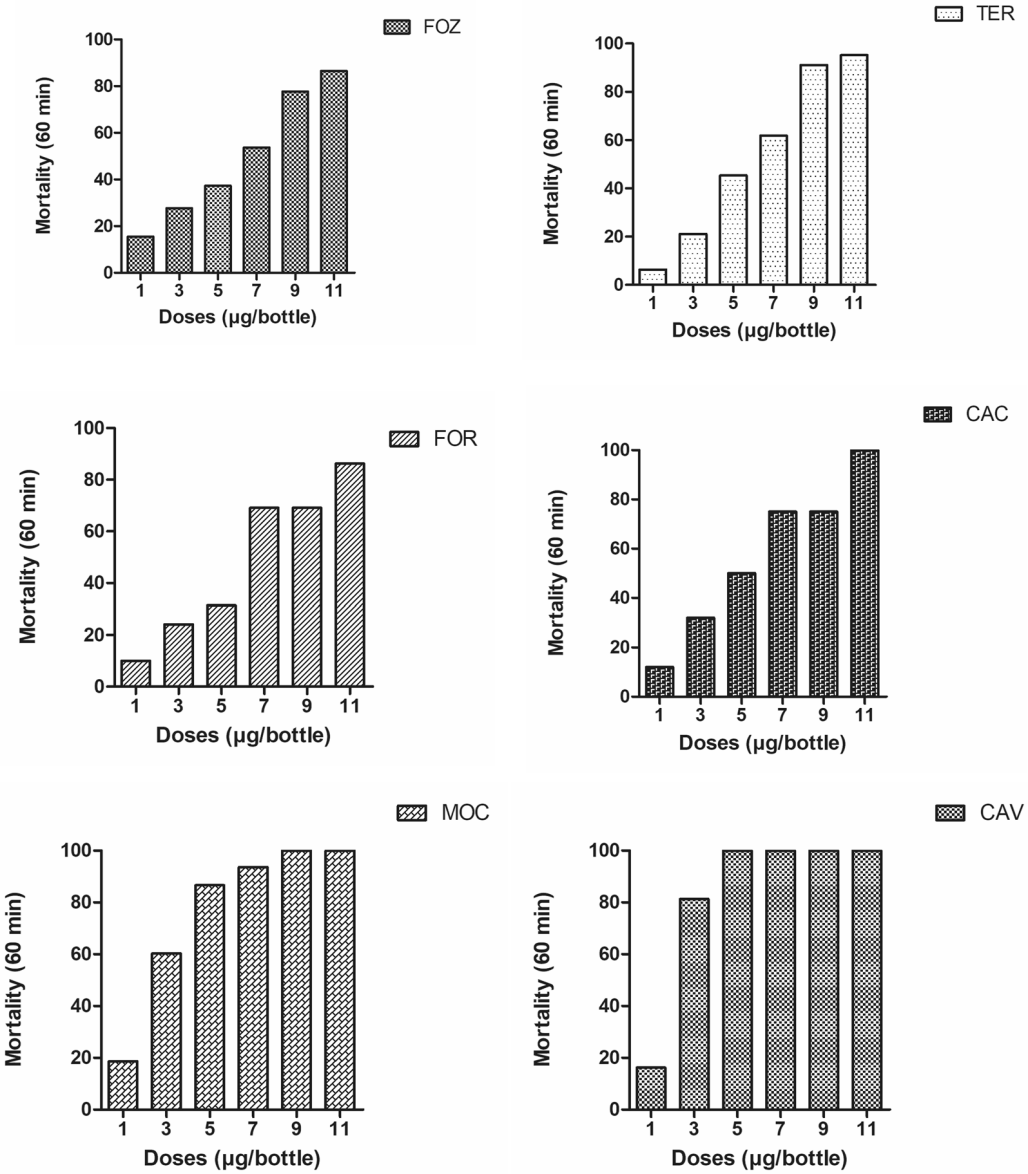
Average mortality of sand fly populations from the different populations for dose responses to the pyrethroid deltamethrin at 60 min of exposure. CAU, Caucaia, Ceará State; CAV, Cavalcante, Goiás State; FOR, Fortaleza, Ceará State; FOZ, Foz do Iguaçu, Paraná State; MOC, Montes Claros, Minas Gerais State; TER, Teresina, Piauí State

The CDC bottle bioassays conducted in different municipalities with varying doses showed that a dose of 5 μg/bottle resulted in 100% mortality for the *Lu. longipalpis* population collected in the municipality of Cavalcante. Subsequent doses maintained this level of mortality, indicating the high susceptibility of this population to deltamethrin. In contrast, *Lu. longipalpis* from Montes Claros exhibited the second-highest mortality rate (86.6%), while the other populations showed lower mortality rates (Fig. 2). Among the six populations exposed to deltamethrin, the Cavalcante sand fly population was the most susceptible, with mortality rates ranging from 30% to 40%.

The LD_50_ of the reference population was 3.50 μg deltamethrin/bottle. The LD_50_ of the field populations ranged from 1.92 to 9.53 μg/bottle. The population from Caucaia had the highest LD_50_ value at 9.53 μg/bottle, while the population from Cavalcante had the lowest value at 1.92 μg/bottle. The populations of *Lu. longipalpis* in Foz do Iguaçu and Caucaia exhibited significant differences in LD_50_ when compared to the reference population (LRS). These results are shown in Table 3, where the confidence limits overlapped at the 95% level. The LD_95_ of the reference population was 12.8 μg/bottle, while the LD_95_ range of the field populations was 4.33 to 53.6 μg/bottle. For LD_95_, the populations of Caucaia and Cavalcante exhibited significant differences when the confidence limits overlapped at the 95% confidence level. The RR_50_ values ranged from 2.27 to 0.54, indicating low levels of resistance for the analyzed populations. The RR_95_ also showed a range of 4.18 to 0.33 (Table 3).

**Table 3:** Population distribution, number of specimens per sample, slope, 50% and 95% lethal doses and resistance ratios of *Lu. longipalpis* populations exposed to the pyrethroid deltamethrin in CDC bottle bioassays in 2023

State	Population	*N*	Slope	LD_50_ (95% CI)^a^	LD_95_ (95% CI)^a^	RR_50_^b^	RR_95_^b^
Bahia	LRS (reference population)	420	2.93	3.50 (1.38–5.73)	12.8 (7.29–12.2)	–	–
Ceará	Caucáia	247	2.19	9.53 (7.09–36.1)	53.6 (20.9–50.1)	2.27	4.18
Paraná	Foz do Iguaçu	325	4.86	7.12 (6.36–7.81)	15.5 (12.9–21.3)	2.03	1.21
Ceará	Fortaleza	337	3.64	6.21(4.75–8.03)	17.5 (9.59–36.2)	1.77	1.36
Piauí	Teresina	258	4.75	5.39 (4.73–5.98)	11.9 (10.2–15.4)	1.54	0.92
Minas Gerais	Montes Claros	279	3.53	2.67 (2.04–3.18)	7.80 (6.18–11.8)	0.76	0.61
Goiás	Cavalcante	328	4.66	1.92 (1.53–2.27)	4.33 (3.57–5.78)	0.54	0.33

^*CI*Confidence interval^

^a^Concentration of insecticide that kills 50% (LD_50_) and 95% (LD_95_) of the samples studied

^b^Resistance ratio calculated using the LD_50_ and 95% LD_95_ of the field populations compared to those of the susceptible reference population

**Fig. 3:**
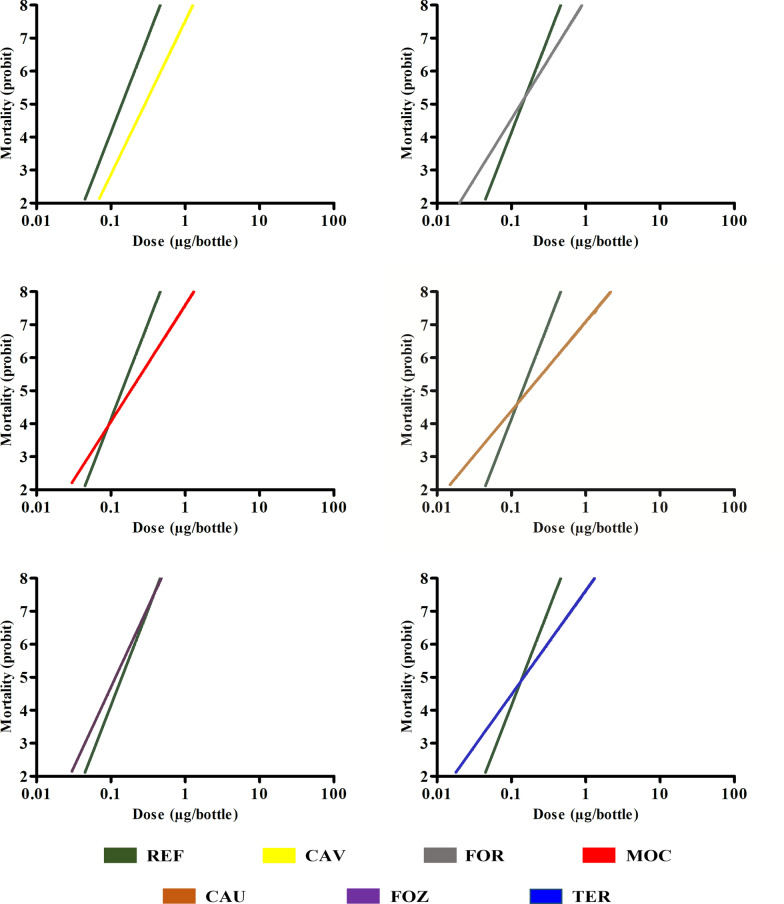
The mortality curve of *Lu. longipalpis* populations, represented on a logarithmic scale, on the insecticide deltamethrin. The reference population (REF) is represented by the laboratory population from Jacobina (green), while the populations from Cavalcante (CAV; yellow), Fortaleza (FOR; gray), Montes Claros (MOC; red), Caucaia (CAU; brown), Foz do Iguaçu (FOZ; purple) and Teresina (TER; blue) are field populations

When compared to the reference population (Fig. 3), the angular coefficient values of the sand fly populations from the municipalities of Teresina and Caucaia showed less homogeneity and a higher frequency of individuals with resistance alleles. In contrast, *Lu. longipalpis* from Foz do Iguaçu and Cavalcante exhibited angular coefficient patterns similar to the reference population. The populations of *Lu. longipalpis* from Fortaleza and Montes Claros exhibited greater heterogeneity compared to the reference population. This suggests the possibility of selecting resistant individuals in the event of insecticide pressure, as shown in Fig. 3.

## Discussion

In this study we assessed the susceptibility profile of six sand fly populations collected in areas where dog collars are used to control VL. A diagnostic dose for the *Lu. longipalpis* species was established using bioassays with bottles as recommended by the excluded the US Centers for Disease Control and Prevention (CDC). The results showed that *Lu. longipalpis* field populations from three sampling locations where insecticide-impregnated dog collars were used for VL control were susceptible to deltamethrin. These results contribute to sand fly resistance surveillance and highlight the need for a better understanding of the resistance mechanisms of *Lu. longipalpis* in areas where impregnated dog collars have been widely used. *Lu. longipalpis* sensu lato is a complex of phlebotomine sand fly species. Our phylogenetic analyses showed that morphologically similar populations of *Lu. longipalpis* collected in Teresina (1S) and Santarém (1S) showed a high degree of genetic divergence. This work highlights the susceptibility responses of *Lu. longipalpis* through CDC bottle bioassays, but there is a need for future studies with genetically different populations to better understand susceptibility within the longipalpis complex [43, 44].

The LD_99_ for the reference population of *Lu. longipalpis* was calculated at 21.9 μg/bottle; thus the DD was 1 × LD_99_ = 21.9 μg/bottle. The results of our study indicated that the DD obtained differed from those reported for *Lu. longipalpis* populations in Colombia, where the DD was 10 μg/bottle for deltamethrin [45, 46]. A recent multi-center laboratory study published the ‘Standard Operating Procedures' for testing sand fly resistance to insecticides in the WHO bottle bioassay and tube test; this study recommends using non-blood-fed female adults aged 3–7 days [47]. For WHO tube tests with deltamethrin, a DD of 0.05% was recommended. However, populations of *Lu. longipalpis* from Colombia and Brazil showed differences in the LD_99_. These differences were explained by variations in genetic structure, such as the presence of sister species and/or differences in the degree of exposure of field populations to insecticides before being colonized. Chaubey et al. [48] also noted that *Ph. argentipes* had varying DD depending on the location and type of insecticide studied. These results emphasize the significance of using a locally estimated DD from a population of *Lu. longipalpis* to enable more precise comparisons of the susceptibility of various field populations.

The population of *Lu. longipalpis* from Caucaia was classified as resistant and possibly resistant when exposed to DD of 21.9 and 30 μg/bottle, respectively. Additionally, the Fortaleza and Montes Claros populations were classified as possibly resistant at a DD of 21.9 μg/bottle. The Teresina population showed possible resistance at a dose of 30 ug/bottle. Impregnated collars were implemented in the municipality of Caucaia in April 2022, with a total of 675 dogs collared in two cycles. In Fortaleza, the collars were implemented in July 2021, with a total of 68,000 dogs using them. Impregnated collars were implemented in the municipality of Teresina in December 2021, with a total of 11,021 dogs collared in three cycles. In Montes Claros, the collars were implemented in July 2021, with a total of 10,949 dogs collared in three cycles. In our study, the sand fly populations from these municipalities had lower mortality rates for *Lu. longipalpis*. This may have influenced the results, as the sand flies faced insecticide pressure for longer due to the use of collars and residual treatments, both using pyrethroid insecticides. Systematic review studies conducted by Rocha et al. [16] and Balaska et al. [17] on the susceptibility status of sand fly populations in the New and Old World revealed that *Ph. argentipes* and *Ph. papatasi* exhibit resistance to various classes of insecticides. However, the susceptibility status of *Lutzomyia* species remains unclear. In a previous study, *Lu. longipalpis* exhibited susceptibility when exposed to various DD and different insecticides [16]. This contrasts with the results obtained in our research, which detected resistance to deltamethrin in field populations of *Lu. longipalpis*.

The populations of *Lu. longipalpis* in Foz do Iguaçu, Montes Claros, Cavalcante and Teresina, which were exposed to 21.9 μg/bottle of deltamethrin, showed high susceptibility to deltamethrin. Falcão et al. [15] reported a high susceptibility of *Lu. longipalpis* populations to this insecticide, similar to observations by Mazzarri et al. [49] in field populations exposed to various classes of insecticide, including the pyrethroid deltamethrin. In Brazil, Rocha et al. [24] evaluated the susceptibility of four field populations of *Lu. longipalpis* to alpha-cypermethrin using CDC bottle bioassays, and found all populations to be highly susceptible. After 60 min of exposure at a DD of 30 μg/bottle, only the populations of Montes Claros and Cavalcante reached 100% mortality. It is important to highlight that the concentrations of 3 μg/bottle to the highest concentration of 11 μg/bottle yielded high mortality rates from both field populations. The Cavalcante population showed 100% mortality after only 30 min of exposure to doses of 5, 7, 9 and 11 μg/bottle. However, the susceptible reference population achieved 100% mortality only after 60 min of exposure to a dose of 11 μg/bottle. Of the municipalities included in the study, those of Montes Claros and Cavalcante had a relatively shorter experience with the insecticide dog collar as the latter was introduced in May 2023 and August 2023, respectively. In Montes Claros, 419 dogs were collared, while in Cavalcante, 359 dogs were collared. Therefore, the population of *Lu. longipalpis* collected in Cavalcante had been exposed to insecticides for a shorter period of time and were collected in a more preserved environment, as the area is protected by the Kalunga Community. These variables may explain why this population is more susceptible, which could be useful as a reference for future studies on sand fly susceptibility to insecticides.

Based on the results of this study, we propose that the *Lu. longipalpis* population from Cavalcante is a potential LRS due to its 100% mortality rate at a 5 μg/bottle dose and lower LD_50_ and LD_95_ values compared to the LRS used in this study. González et al. [25] also identified that *Lu. longipalpis* from the laboratory presented greater tolerance to deltamethrin and lambdacyhalothrin compared to populations that were collected in the field in the municipality of Araçatuba, São Paulo State, Brazil. In addition, *Lu. longipalpis* from Gruta da Lapinha, Minas Gerais State was found to be a laboratory reference strain in susceptibility studies with pyrethroids [26, 27]. In general, all the populations exhibited low levels of resistance when compared to the LRS, as the RR_50_ ranged from 2.27 (Caucaia) to 0.54 (Cavalcante). The results obtained in the present study are consistent with those reported by Bidabadi et al. [50], who estimated a RR_50_ of 2.52 for the species *Ph. papatasi* in susceptibility experiments with WHO tubes. The populations from Teresina, Caucaia, Montes Claros and Fortaleza exhibited greater heterogeneity than the LRS, indicating a higher likelihood of selecting resistant individuals. Conversely, the population from Cavalcante, Goiás State displayed the greatest homogeneity. The slope enables us to draw conclusions about the level of genetic variability in a population, which indicates a progression of resistance. Populations with low genetic variability are less likely to change their resistance ratio, while populations with higher genetic variability are more likely to change their resistance ratio in response to the insecticide used over time [51].

There are a number of limitations to this study. First, an insufficient number of sand flies were available to carry out the bioassays. Second, sample loss occurred during the identification process. A comparison of the populations in Teresina, Caucaia, Montes Claros and Fortaleza with the LRS revealed greater heterogeneity, indicating a greater probability of selection of resistant individuals. Furthermore, research is required to analyze penetration, metabolic and genetic resistance to gain a deeper understanding of the dynamics of selecting insecticide-tolerant populations. A systematic review of the susceptibility status of sand fly populations in the New and Old World was conducted by Rocha et al. [16] and Balaska et al. [17], revealing that most resistance bioassays for sand flies used WHO tube kits, yet the standardization of DD and/or an LRS was not consistently applied. In this context, we wish to draw attention to the use of CDC bottles, which are inexpensive and easy to transport and handle. Furthermore, we highlight the use of field-collected populations in the experiments, as recommended by the WHO [47]. We believe it is important to overcome these limitations in future studies to evaluate the susceptibility of field populations of *Lu. longipalpis* to insecticides in areas where insecticide-impregnated dog collars are used to control VL. This is important because insecticide-impregnated dog collars can reduce the risk of VL in dogs [52] and provide a significant level of protection against VL in humans [53]. Therefore, insecticide-impregnated dog collars could be a viable alternative for inclusion as a public health measure to control VL if sand flies are susceptible to insecticide-impregnated dog collars.

## Conclusions

This study established a DD for *Lu. longipalpis* using the CDC bottle bioassay. We found that *Lu. longipalpis* populations in three Brazilian states where insecticide-impregnated dog collars were used for VL control were susceptible to deltamethrin. In contrast, one population in Ceará State was classified as resistant to deltamethrin. These results contribute to sand fly resistance surveillance and highlight the need for a better understanding of the resistance mechanisms of *Lu. longipalpis* in areas where insecticide-impregnated dog collars have been widely used.

## Data Availability

All data are available in the article.

## References

[1] Scarpini S, Dondi A, Totaro C, Biagi C, Melchionda F, Zama D, et al. Visceral leishmaniasis: epidemiology, diagnosis, and treatment regimens in different geographical areas with a focus on pediatrics. Microorganisms. 2022;10:13–1. 10.3390/microorganisms10101887 PMC9609364 36296164

[2] Costa CHN, Chang KP, Costa DL, Cunha FVM. From infection to death: an overview of the pathogenesis of visceral leishmaniasis. Pathogens. 2023;12:969. 37513817 10.3390/pathogens12070969 PMC10384967

[3] Martins IM, Silva JS, Campos DK, Oliveira RS, Silva PL, Carvalho SF, et al. Visceral leishmaniasis: historical series of hospitalized patients and correlation with climate in an endemic area in Minas Gerais, Brazil. J Bras Patol Med Lab. 2021;57:7–1.

[4] Franssen SU, Durrant C, Stark O, Moser B, Downing T, Imamura H, et al. Global genome diversity of the *Leishmania donovani* complex. Elife. 2020;25:44–51. 10.7554/eLife.51243 PMC7105377 32209228

[5] Pan American Health Organization (Organização Pan-Americana da Saúde). Leishmanioses: informe epidemiológico das Américas. Núm. 12 (Dezembro de 2023). 2024. https://iris.paho.org/handle/10665.2/59170. Accessed 10 Apr 2024.

[6] Rangel EF, Vilela ML. Lutzomyia longipalpis (Diptera, Psychodidae, Phlebotominae) and urbanization of visceral leishmaniasis in Brazil. Cad Saude Publica. 2008;24:2948–52. 19082287 10.1590/s0102-311x2008001200025

[7] Brasil. Ministério da Saúde. Sistema de Informação de Agravos de Notificação (SINAN). 2022. http://portalsinan.saude.gov.br/. Accessed 10 May 2024.

[8] Volpedo G, Pacheco-Fernandez T, Bhattacharya P, Oljuskin T, Dey R, Gannavaram S, et al. Determinants of innate immunity in visceral leishmaniasis and their implication in vaccine development. Front Immunol. 2021;12:748325. 34712235 10.3389/fimmu.2021.748325 PMC8546207

[9] Burza S, Croft SL, Boelaert M. Leishmaniasis. Lancet. 2018;392:951–70. 30126638 10.1016/S0140-6736(18)31204-2

[10] Costa DC, Bermudil PM, Rodas LC, Nunes MC, Hiramoto RM. Human visceral leishmaniasis and relationship with vector and canine control measures. Rev Saúde Pública. 2018;52:11–21. 30484481 10.11606/S1518-8787.2018052000381 PMC6280620

[11] Brasil Ministério da Saúde, Secretaria de Vigilância em Saúde, Departamento de Vigilância Epidemiológica. Manual de vigilância e controle da leishmaniose visceral. Série A. Normas e Manuais Técnicos. Brasília: Editora do Ministério da Saúde; 2014.

[12] Amóra SS, Bevilaqua CM, Feijó FM, Alves D. Control of phlebotomine (Diptera: Psychodidae) leishmaniasis vectors. Neotrop Entomol. 2009;38:303–10. 19618043 10.1590/s1519-566x2009000300001

[13] Alexander B, Maroli M. Control of phlebotomine sandflies. Med Vet Entomol. 2003;17:18–21. 10.1046/j.1365-2915.2003.00420.x 12680919

[14] Teodoro U, Galati EAB, Kühl JB, Lozovei AL, Barbosa OC. Controle de flebotomíneos com DDT, em área endêmica de leishmaniose tegumentar no Estado do Paraná, sul do Brasil. Braz Arc Biol Tech. 1998;41:359–64.

[15] Falcão AR, Pinto CT, Gontijo CMF. Suscetibility of *Lutzomyia longipalpis* to deltametrin. Mem Inst Oswaldo Cruz. 1988;83:395–6. 3271938 10.1590/s0074-02761988000300020

[16] Rocha DA, Costa LMD, Pessoa GDC, Obara MT. Methods for detecting insecticide resistance in sand flies: a systematic review. Acta Trop. 2021;213:105747. 33188748 10.1016/j.actatropica.2020.105747

[17] Balaska S, Fotakis EA, Chaskopoulou A, Vontas J. Chemical control and insecticide resistance status of sand fly vectors worldwide. PLoS Negl Trop Dis. 2021;15:23–31. 10.1371/journal.pntd.0009586 PMC8360369 34383751

[18] Dinesh DS, Das ML, Picado A, Roy L, Rijal S, Singh SP, et al. Insecticide susceptibility of *Phlebotomus argentipes* in visceral leishmaniasis endemic districts in India and Nepal. PLoS Negl Trop Dis. 2010;4:859. 10.1371/journal.pntd.0000859 PMC2964302 21049013

[19] Singh R, Das RK, Sharma SK. Resistance of sandflies to DDT in Kala-azar endemic districts of Bihar, India. Bull World Health Organ. 2001;79:793. 11545338 PMC2566489

[20] Amalraj DD, Sivagnaname N, Srinivasan R. Susceptibility of* Phlebotomus argentipes *and* P. papatasi* (Diptera: Psychodidae) to insecticides. J Commun Dis. 1999;31:177–80. 10916614

[21] Bansal SK, Singh KV. Susceptibility status of *Phlebotomus papatasi* and *Sergentomyia punjabaensis* (Diptera:Psychodidae) to some insecticides in district Bikaner (Rajasthan). J Commun Dis. 1996;28:28–32. 8778177

[22] Kaul SM, Wattal BL, Bhatnagar VN, Kk Mathur. Preliminary observations on the susceptibility status of *Phlebotomus argentipes* and *P. papatasi* to DDT in two districts of North Bihar (India). J Commun Dis. 1978;10:208–11.

[23] Arzamani K, Vatandoost H, Rassi Y, Abai MR, Akhavan AA, Alavinia M, et al. Susceptibility status of wild population of *Phlebotomus sergenti* (Diptera: Psychodidae) to different imagicides in an endemic focus of cutaneous leishmaniasis in northeast of Iran. J Vector Borne Dis. 2017;54:282–6. 29097645 10.4103/0972-9062.217621

[24] Rocha DA, Andrade AJ, Moura LR, Figueiredo NG, Pessoa GCD, Obara MT. Susceptibility of phlebotomine sandflies (Diptera: Psychodidae) collected in the field, to alpha‑cypermethrin in four municipalities endemic to leishmaniasis. Rev Inst Med Trop São Paulo. 2020;62:4–1. Accessed 11 Apr 2024. 10.1590/S1678-9946202062038 PMC7274764 32520213

[25] González MA, Bell MJ, Bernhardt SA. Susceptibility of wild- caught *Lutzomyia longipalpis* (Diptera: Psychodidae) sand flies to insecticide after an extended period of exposure in western São Paulo Brazil. Parasit Vectors. 2019;12:9–1. 30871639 10.1186/s13071-019-3364-4 PMC6419423

[26] Pessoa GC, Lopes JV, Rocha MF, Pinheiro LC, Rosa AC, Michalsky ÉM, et al. Baseline susceptibility to alpha-cypermethrin in *Lutzomyia longipalpis* (Lutz & Neiva, 1912) from Lapinha Cave (Brazil). Parasit Vectors. 2015;17:8–1. 10.1186/s13071-015-1076-y PMC4573933 26381242

[27] Alexander B, Barros VC, de Souza SF, Barros SS, Teodoro LP, Soares ZR, et al. Susceptibility to chemical insecticides of two Brazilian populations of the visceral leishmaniasis vector *Lutzomyia longipalpis* (Diptera: Psychodidae). Trop Med Int Health. 2009;14:1272–7. 19772549 10.1111/j.1365-3156.2009.02371.x

[28] Alves EB, Figueiredo FB, Rocha MF, Castro MC, Werneck GL. Effectiveness of insecticide-impregnated collars for the control of canine visceral leishmaniasis. Prev Vet Med. 2020;182:7–1. 10.1016/j.prevetmed.2020.105104 32759025

[29] Silva RA, Andrade AJ, Quint BB, Raffoul GS, Werneck GL, Rangel EF. Effectiveness of dog collars impregnated with 4% deltamethrin in controlling visceral leishmaniasis in *Lutzomyia longipalpis* (Diptera: Psychodidade: Phlebotominae) populations. Mem Inst Oswaldo Cruz. 2018;113:9–1. 29590235 10.1590/0074-02760170377 PMC5868867

[30] Kazimoto TA, Amora SSA, Figueiredo FB, Magalhães JME, Freitas YBN, Sousa MLR, et al. Impact of 4% Deltamethrin-Impregnated dog collars on the prevalence and incidence of canine visceral leishmaniasis. Vector Borne Zoonotic Dis. 2018;18:356–63. 29683394 10.1089/vbz.2017.2166

[31] David JR, Stamm L, Bezerra H, Souza R, Killick KR, Lima J. Deltamethrin- impregnated dog collars have a potent anti-feeding and insecticidal effect on *Lutzomyia longipalpis* and *Lutzomyia migonei*. Mem Inst Oswaldo Cruz. 2001;9:839–47. 10.1590/s0074-02762001000600018 11562713

[32] Brasil. Ministério da Saúde. Guia de vigilância em saúde. Ministério da Saúde. Brasília: Ministério da Saúde; 2021.

[33] Denlinger DS, Creswell JA, Anderson JL, Reese CK, Bernhardt SA. Diagnostic doses and times for *Phlebotomus papatasi* and *Lutzomyia longipalpis* sand flies (Diptera: Psychodidae: Phlebotominae) using the CDC bottle bioassay to assess insecticide resistance. Parasit Vectors. 2016;9:11–1. 27083417 10.1186/s13071-016-1496-3 PMC4833940

[34] WHO. Standard operating procedures for testing insecticide susceptibility of adult mosquitoes in WHO bottle bioassays. 2022. https://www.who.int/publications/i/item/9789240043770. Accessed 10 Apr 2024.

[35] Rocha DA, Costa LM, Pessoa GCD, Obara MT. Standardization of laboratory bioassays for the study of *Lutzomyia longipalpis* (Diptera: Psychodidae) susceptibility to pyrethroid insecticides. In: Oliveira J, Alevi KCC, Camargo LM, Meneguetti DUO, editors. Atualidades em medicina tropical no Brasil: Vetores. Rio Branco: Strictu Sensu; 2023. p. 192–202.

[36] WHO. monitoring and managing insecticide resistance in aedes mosquito populations. Interim guidance for entomologists. 2016. https://www.who.int/publications/i/item/WHO-ZIKV-VC-16.1. Accessed 10 April 2024.

[37] Forattini OP. Gênero Lutzomyia. In: Forattini OP, editor. Entomologia Médica. São Paulo: 1973. Editora Edgard Blucher. p.206–387.

[38] Galati EAB. Apostila de bioecologia e identificação de Phlebotominae (Diptera, Psychodidae)–Departamento de Epidemiologia. São Paulo: Faculdade de Saúde Pública da USP; 2015.

[39] Rocha DA, Almeida MR, Batista JAS, Andrade AJ. LutzoDex™—a digital key for Brazilian sand flies (diptera, phlebotominae) within an android app. Zootaxa. 2019;4688:3. 10.11646/zootaxa.4688.3.4 31719438

[40] Marcondes CB. A proposal of generic and subgeneric abbreviations for phlebotomine sandflies (Diptera: Psychodidae: Phlebotominae) of the world. Entomol News. 2007;118:351–6.

[41] Litchfield JT Jr, Wilcoxon F. A simplified method of evaluating dose-effect experiments. J Pharmacol Exp Ther. 1949;96:99–113. 18152921

[42] LeOra Software Company. PoloPlus. Polo for Windows computer program version 20. Reading, Berkshire: LeOra Software; 2005.

[43] Sousa-Paula LC, Pessoa FAC, Otranto D, Dantas-Torres F. Beyond taxonomy: species complexes in New World phlebotomine sand flies. Med Vet Entomol. 2021;35:267–83. 33480064 10.1111/mve.12510

[44] Sousa-Paula LC, da Silva LG, da Silva Junior WJ, Figueirêdo Júnior CAS, Costa CHN. Genetic structure of allopatric populations of Lutzomyia longipalpis sensu lato in Brazil. Acta Trop. 2021. 10.1016/j.actatropica.2021.106031. Accessed 10 Apr 2024. 34224718 10.1016/j.actatropica.2021.106031

[45] Marceló C, Cabrera OL, Santamaria E. Discriminating concentrations for three insecticides used in public health in a *Lutzomyia longipalpis* experimental strain from Colombia. Biomedica. 2014;34:630–624. 10.1590/S0120-41572014000400016 25504252

[46] Santamaría E, Munstermann LE, Ferro C. Approximation to the CDC method to determine insecticide susceptibility in leishmaniasis vectors. Biomedica. 2003;23:121–115. 12696404

[47] WHO. Determining discriminating concentrations of insecticides for monitoring resistance in sand flies: report of a multi-centre laboratory study and WHO expert consultations. 2022. https://www.who.int/publications/i/item/9789240064416. Accessed 10 Apr 2024.

[48] Chaubey R, Shukla A, Kushwaha AK, Tiwary P, Kumar Singh S, Hennings S, et al. Assessing insecticide susceptibility, diagnostic dose and time for the sand fly *Phlebotomus argentipes*, the vector of visceral leishmaniasis in India, using the CDC bottle bioassay. PLoS Negl Trop Dis. 2023;17:14–21. 10.1371/journal.pntd.0011276 PMC10202287 37163529

[49] Mazzarri MB, Feliciangeli MD, Maroli M, Hernandez A, Bravo A. Susceptibility of *Lutzomyia longipalpis* (Diptera: Psychodidae) to selected insecticides in an endemic focus of visceral leishmaniasis in Venezuela. J Am Mosq Control Assoc. 1997;13:335–41. 9474559

[50] Bidabadi L, Oshaghi MA, Enayati AA, Akhavan AA, Zahraei-Ramazani AR, Yaghoobi-Ershadi MR, et al. Molecular and Biochemical Detection of Insecticide Resistance in the Leishmania Vector, *Phlebotomus papatasi* (Diptera: Psychodidae) to Dichlorodiphenyltrichloroethane and Pyrethroids. Central Iran J Med Entomol. 2022;59:1347–54. 35595289 10.1093/jme/tjac031

[51] Obara MT, Otrera VMG, Gonçalves RG, Santos JP, Santalucia M, Rosa JR, et al. Monitoring the susceptibility of *Triatoma sordida* Stål, 1859 (Hemiptera: Reduviidae) to deltamethrin insecticide, in Central Western Brazil. Rev Soc Bras Med Trop. 2011;44:206–12. 21556492 10.1590/s0037-86822011005000004

[52] Yimam Y, Mohebali M. Effectiveness of insecticide-impregnated dog collars in reducing incidence rate of canine visceral leishmaniasis: A systematic review and meta-analysis. PLoS ONE. 2020;15:e0238601. 32881961 10.1371/journal.pone.0238601 PMC7470253

[53] Courtenay O, Bazmani A, Parvizi P, Ready PD, Cameron MM. Insecticide-impregnated dog collars reduce infantile clinical visceral leishmaniasis under operational conditions in NW Iran: A community-wide cluster randomised trial. PLoS Negl Trop Dis. 2019;13:e0007193. 30830929 10.1371/journal.pntd.0007193 PMC6417739

